# How equipped are pharmacists for pharmacogenomics?: A cross-sectional study on knowledge, attitudes, and implementation practices in Saudi Arabia

**DOI:** 10.1097/MD.0000000000042240

**Published:** 2025-04-25

**Authors:** Mohammed S. Alharthi

**Affiliations:** a Department of Clinical Pharmacy, College of Pharmacy, Taif University, Taif, Saudi Arabia.

**Keywords:** clinical implementation, genetic testing, personalized medicine, pharmacists’ knowledge, pharmacogenomics

## Abstract

Pharmacogenomics, the study of genetic influences on drug response, advances personalized medicine by tailoring therapy to individual genetic profiles, reducing adverse effects and optimizing efficacy. Pharmacists, as accessible healthcare providers, are well-positioned to facilitate the integration of pharmacogenomics into clinical practice. This study assesses Saudi pharmacists’ knowledge, attitudes, and practices regarding pharmacogenomics and identifies key barriers and facilitators affecting their readiness. A cross-sectional survey was conducted between July and October 2024 among 426 licensed pharmacists across various practice settings in Saudi Arabia: a validated, structured questionnaire assessed demographics, pharmacogenomics knowledge, attitudes, and implementation practices. Snowball sampling facilitated participant recruitment. Descriptive statistics summarized the findings, and Chi-square tests were applied to examine associations between socio-demographic variables and pharmacogenomics-related responses. Among 426 participants, while most pharmacists recognized the value of pharmacogenomics, knowledge gaps were notable, particularly in interpreting genetic tests and applying clinical recommendations. Only 52.3% received pharmacogenomics training, mainly through university courses, and 40.6% had never consulted pharmacogenomics resources in practice. Key barriers included limited access to genetic testing (74.2%) and lack of reimbursement (64.5%). Socio-demographics, such as age and practice setting, significantly impacted knowledge and attitudes. Saudi pharmacists face considerable barriers to pharmacogenomics readiness, including knowledge gaps, limited access to genetic testing, and insufficient institutional support. Addressing these challenges requires targeted education, structured policy initiatives, and enhanced resource availability to facilitate the effective integration of pharmacogenomics into pharmacy practice. Strengthening pharmacists’ competencies in this field will be essential to optimizing patient care and advancing precision medicine in Saudi Arabia.

## 1. Introduction

Pharmacogenomics, a specialized field within personalized medicine, investigates the influence of genetic variations on drug response and facilitates the optimization of medication regimens based on individual genetic profiles.^[1]^ This approach enhances therapeutic efficacy, minimizes adverse drug reactions, and improves patient safety and treatment outcomes.^[2]^ By leveraging pharmacogenomic insights, healthcare professionals can transition from conventional “one-size-fits-all” prescribing paradigms toward a more tailored approach, wherein drug selection and dosing are individualized.^[3]^ This advancement holds promise in therapeutic areas where inter-individual variability in drug metabolism and response is well-documented, including oncology, cardiology, psychiatry, and infectious diseases.^[4]^ Pharmacists, as accessible healthcare professionals with expertise in pharmacotherapy and medication management, are well-positioned to facilitate the clinical implementation of pharmacogenomics.^[5]^ Their role extends beyond medication dispensing to encompass the interpretation of genetic test results, formulation of pharmacogenomics-guided therapeutic recommendations, provision of patient education, and interdisciplinary collaboration to support the integration of genetic data into clinical decision-making.^[6]^ However, despite the recognized potential of pharmacists in advancing pharmacogenomics-informed precision medicine, its integration into routine pharmacy practice remains suboptimal due to multiple challenges, including knowledge deficits, inadequate formal education, and restricted access to essential pharmacogenomic resources^[7]^ Recent studies from North America and Europe have highlighted that while pharmacists recognize the significance of pharmacogenomics, they often report insufficient training in interpreting genetic test results and incorporating them into medication therapy management. For instance, a 2022 survey of UK pharmacy professionals revealed that only 13% had received formal genomics training, and only 8% felt prepared to use genomic testing in their practice.^[8]^ In North America, research indicates that while pharmacy programs have incorporated pharmacogenomics into their curricula, students often feel unprepared to apply this knowledge clinically due to varying content and teaching methods.^[9]^ In the Middle Eastern and Arab regions, the implementation of pharmacogenomics faces multiple barriers, including limited awareness, restricted access to genetic testing, and concerns regarding ethical, legal, and financial issues.^[10]^ The adoption of pharmacogenomics remains inconsistent, with healthcare professionals, including pharmacists and physicians, frequently reporting inadequate knowledge and training in this domain. For instance, a study assessing healthcare professionals in Kuwait found that only 8.9% had received education or training in pharmacogenomics, with a mean knowledge score of 45% among respondents.^[11]^ Similarly, research in the United Arab Emirates identified cost, lack of training or education, and insufficient insurance coverage as top barriers to implementing genetic testing.^[12]^ While several studies have explored pharmacogenomics awareness among healthcare professionals in Saudi Arabia, there is a notable lack of focused assessments specifically evaluating pharmacists’ knowledge, attitudes, and practical engagement with pharmacogenomics. For instance, a cross-sectional study conducted among community pharmacists in Riyadh revealed that only 33% of participants demonstrated good knowledge of pharmacogenomics and genetics, indicating a significant knowledge gap in this area.^[13]^ Similarly, research assessing hospital pharmacists in Saudi Arabia found that only 30% had received formal training in pharmacogenomics, with respondents reporting a moderate to low level of knowledge.^[14]^ These findings underscore the need for targeted research that focuses on pharmacists to effectively integrate pharmacogenomics into clinical practice, with a primary emphasis on well-established and clinically actionable pharmacokinetic genetic variations. While recognizing that pharmacodynamic genetic associations may currently have less frequent clinical relevance due to their rarity, enhancing pharmacist competencies in more common pharmacokinetic-related genetic variations remains critical for optimizing patient care.^[1]^ This study is unique because it provides the first comprehensive assessment of pharmacists’ knowledge, attitudes, and practices (KAP) in pharmacogenomics within Saudi Arabia. It offers empirical insights into pharmacists’ preparedness and the foundational steps needed for the future integration of pharmacogenomics into routine practice. Furthermore, this study examines key socio-demographic variables influencing pharmacists’ engagement with pharmacogenomics, a factor often overlooked in previous research, by identifying how factors such as age, years of experience, and type of practice setting impact pharmacists’ competency and attitudes, this study provides novel insights into the professional determinants of pharmacogenomics adoption. Therefore, this study aims to explore pharmacists’ knowledge, attitudes, and implementation practices related to pharmacogenomics in Saudi Arabia, specifically investigating potential barriers such as access to genetic testing, institutional readiness, and economic constraints, including reimbursement policies for pharmacogenomic services.

## 2. Methods

### 2.1. Study design

This cross-sectional study was conducted among licensed pharmacists from diverse practice settings in Saudi Arabia to assess their KAP toward pharmacogenomics. Data collection occurred from July to October 2024, and pharmacists with active registration were included in the study. Pharmacists were recruited from various practice settings, including community pharmacies, hospital pharmacies, clinical pharmacy departments, research institutions, and academic settings. This diverse representation enabled a comprehensive assessment of pharmacogenomics engagement across various pharmacy roles. Those who were not currently practising were excluded to ensure data accuracy.

### 2.2. Sampling and sample size

A snowball sampling approach was employed to capture diverse perspectives across various pharmacy practice settings, including community pharmacies, hospital pharmacies, clinical pharmacy departments, research institutions, and academic settings. The sample size was determined using Cochran formula for calculating sample size in cross-sectional studies: n = Z² (1 − p)/d².^[15]^ Where n represents the required sample size, Z is the Z-score corresponding to a 95% confidence interval (1.96), p is the anticipated proportion (set at 0.5), and d is the margin of error (set at 0.05). Since no prior prevalence data were available on pharmacists’ KAP toward pharmacogenomics in Saudi Arabia, the proportion was conservatively set at 0.5. This approach is commonly used in survey-based research when population prevalence is unknown, as it yields the most significant sample size, ensuring statistical power and accounting for variability.^[16]^ Substituting these values into the formula required a minimum sample size of 384 participants. The sample size was increased to 426 to enhance response validity and account for potential nonresponse bias. This expansion ensured broader representation across various pharmacy practice settings while maintaining statistical robustness.^[17]^ The decision to increase the sample size also aimed to mitigate the effects of missing data and enhance the reliability of subgroup analyses, particularly in assessing the associations between socio-demographic variables and pharmacists’ KAP regarding pharmacogenomics.

### 2.3. Data collection and piloting

The study questionnaire was developed based on a comprehensive review of published literature on pharmacists’ KAP in pharmacogenomics.^[18–21]^ The initial questionnaire items were adapted from previously validated instruments in studies assessing pharmacogenomics knowledge and implementation among pharmacists and healthcare professionals. The questionnaire was designed in English, the primary language of instruction in Saudi pharmacy education and practice. No translation was required for this study, as all participants were proficient in English, eliminating concerns about linguistic bias or misinterpretation. However, to ensure content validity, the questionnaire underwent expert validation by a panel of specialists in pharmacogenomics, clinical pharmacy, and pharmacy education, who assessed the clarity, relevance, and appropriateness of each item. This expert panel included 3 pharmacogenomics researchers, 2 clinical pharmacists with experience in pharmacogenomics application, and 2 pharmacy education specialists. The validation process involved iterative modifications based on expert feedback, refining ambiguous or redundant questions and ensuring that the questionnaire accurately captured pharmacists’ competencies and perspectives. The questionnaire was structured into 4 key domains, each assessing a specific aspect of pharmacogenomics knowledge, attitudes, and implementation practices:

- Demographic and professional characteristics (7 items) – including age, gender, highest degree, years of experience, and type of pharmacy practice.
- Knowledge of pharmacogenomics (9 items): Assess familiarity with core pharmacogenomics concepts, education sources, and knowledge of pharmacogenomics resources.
- Attitudes toward pharmacogenomics (8 items): evaluate perceived importance, willingness to integrate pharmacogenomics into practice, and confidence in applying pharmacogenomics knowledge.
- Implementation practices and barriers (10 items): assessed current levels of use (if any), frequency of consulting pharmacogenomics resources, and pharmacists perceived institutional and logistical barriers, acknowledging that community pharmacists may report minimal engagement due to resource constraints.

To further ensure internal consistency and feasibility, a pilot study was conducted with ten pharmacists in various practice settings, including community, hospital, and academic pharmacies. The Cronbach alpha for internal reliability was 0.74, indicating acceptable internal consistency across the questionnaire domains. The pilot participants provided qualitative feedback on readability and ease of response, which led to minor refinement before the survey was finalized.

### 2.4. Data analysis

The data collected were analyzed using IBM SPSS Statistics 25.0 and structured into 2 primary stages. In the first stage, descriptive statistics were used to summarize participant characteristics and responses, with means and standard deviations calculated for continuous variables and frequencies and percentages reported for categorical variables. A categorization approach assessed pharmacists’ KAP levels. The knowledge, attitude, and practice scores were calculated based on responses to relevant items, with each response assigned a corresponding numerical value. The total scores were then classified into poor, moderate, and sound levels using modified Bloom cutoff criteria, a widely used method in KAP studies.^[22,23]^ The classification was as follows:

- Good Knowledge/Attitude/Practice: ≥ 75% of the total score
- Moderate Knowledge/Attitude/Practice: 50% to 74% of the total score
- Poor Knowledge/Attitude/Practice: < 50% of the total score

The categorization allowed for a more structured interpretation of pharmacists’ preparedness in pharmacogenomics. For inferential analysis, Pearson chi-square test was applied to examine associations between socio-demographic factors (e.g., age, years of experience, and type of pharmacy practice) and KAP levels. A *P*-value of <.05 was considered statistically significant. Following the Strengthening the Reporting of Observational Studies in Epidemiology checklist, this study was designed and reported to ensure methodological rigour and transparency.

### 2.5. Ethical considerations

Ethical approval was obtained from Taif University’s Committee for this study, with application number 46–009. Participants were provided with information about the study’s purpose, procedures, risks, and benefits, ensuring informed consent was obtained voluntarily. Confidentiality was strictly maintained, with all responses anonymized and stored securely to safeguard participants’ privacy and adhere to ethical guidelines.

## 3. Results

Table 1 shows that most participants (38%) were aged 30 to 39, followed by 26.3% aged 25 to 29. The sample consisted of 72.5% males. Most participants held a bachelor’s degree in pharmacy (35%), with a notable portion holding a master’s degree (29.6%) or a Doctorate (19.2%). Regarding experience, 33.1% had 0 to 5 years, while 25.1% had 16 to 20 years of experience. Hospital pharmacy was the most common practice setting (37.1%), followed by clinical pharmacy (33.6%). Additionally, 10.1% of participants reported working primarily in research, which included pharmacists engaged in pharmaceutical and clinical research within academic institutions, research centers, and hospital-based research units. These individuals were involved in pharmacogenomics research, drug development, clinical trials, and other pharmaceutical science investigations. The remaining 10.8% of participants were categorized under “Other” practice settings, which included roles in regulatory affairs, the pharmaceutical industry, and administrative positions within healthcare institutions.

**Table 1:** Demographic and professional characteristics of study participants (n = 426).

Characteristic	n	(%)
Age (years)		
From 25 to 29	112	26.3
From 30 to 39	162	38.0
From 40 to 49	102	23.9
Above 50	50	11.7
Gender		
Male	309	72.5
Female	117	27.5
Education level		
Bachelor’s degree in pharmacy	149	35.0
Master’s degree in pharmacy	126	29.6
Doctorate in Pharmacy	82	19.2
Other	69	16.2
Years of experience		
0–5 years	141	33.1
6–10 years	82	19.2
11–15 years	58	13.6
16–20 years	107	25.1
21 years and above	38	8.9
Type of pharmacy practice		
Community Pharmacy	36	8.5
Hospital Pharmacy	158	37.1
Clinical Pharmacy	143	33.6
Research	43	10.1
Other	46	10.8

n = Number of participants, % = Percentage.

Table 2 indicates that familiarity with pharmacogenomics is generally low, with only 17.1% of participants being very familiar and 1.9% extremely familiar. Over half (52.3%) have received formal pharmacogenomics education, primarily through university courses (65%). Understanding key concepts is generally basic, with most participants reporting basic knowledge of genetic variations affecting drug response (52.8%) and the clinical implications of pharmacogenomics (62%). Pharmacogenomics resources are rarely consulted in practice, with 40.6% never using them and 42.3% being unfamiliar with any resources. Additionally, only 27.2% have participated in pharmacogenomics research or projects.

**Table 2:** Pharmacists’ knowledge towards pharmacogenomics (n = 426).

Question	n	(%)
How familiar are you with the term “pharmacogenomics”?		
Not familiar at all	94	22.1
Slightly familiar	128	30.0
Moderately familiar	123	28.9
Very familiar	73	17.1
Extremely familiar	8	1.9
Have you received any formal education or training in pharmacogenomics?		
No	203	47.7
Yes	223	52.3
If yes, please specify the type of education or training (n = 223)		
University course	145	65.0
Continuing education program	28	12.6
Workshop or seminar	17	7.6
Other	33	14.8
How often do you consult pharmacogenomics resources (e.g., databases, guidelines) in your practice?		
Never	173	40.6
Rarely	139	32.6
Occasionally	38	8,9
Frequently	50	11.7
Always	26	6.1
Which of the following pharmacogenomics resources are you familiar with? (n = 426)(k = 508)		
Clinical Pharmacogenomics Implementation Consortium (CPIC) guidelines	45	10.6
Pharm GKB database	45	10.6
FDA drug labeling	148	34.7
European Medicines Agency (EMA) guidelines	18	4.2
Other	72	16.9
I am not familiar with any pharmacogenomics resources	180	42.3
Have you ever participated in any pharmacogenomics research or projects?		
No	310	72.8
Yes	116	27.2
Rate your understanding of the following pharmacogenomics concepts:		
*Genetic variations affecting drug response*		
No Understanding	77	18.1
Basic Understanding	225	52.8
Moderate Understanding	59	13.8
Complete Understanding	65	15.3
Pharmacokinetics and pharmacodynamics		
No Understanding	110	25.8
Basic Understanding	97	22.8
Moderate Understanding	103	24.2
Complete Understanding	116	27.2
Clinical implications of pharmacogenomics		
No Understanding	98	23.0
Basic Understanding	264	62.0
Moderate Understanding	34	8.0
Complete Understanding	30	7.0
Genetic testing and interpretation		
No Understanding	126	29.6
Basic Understanding	222	52.1
Moderate Understanding	57	13.4
Complete Understanding	21	4.9
Ethical and legal issues in pharmacogenomics		
No Understanding	144	33.8
Basic Understanding	185	43.4
Moderate Understanding	85	20.0
Complete Understanding	12	2.8

FDA = Food and Drug Administration, n = number of participants, PharmGKB = pharmacogenomics knowledge base, k = number of responses in multiple responses questions.

Table 3 highlights that most pharmacists consider pharmacogenomics important in personalized medicine, with 43.9% rating it as slightly essential and 23.2% as extremely important. Most (58.7%) are only slightly confident in applying it clinically. Key concerns include lack of knowledge and training (68.5%) and the cost of genetic testing (61%). While 61% believe pharmacy practice is unprepared for pharmacogenomics, 47.2% remain neutral on whether the cost of testing is justified. Only 35% agree that pharmacogenomics testing should be routine, and a minority (10.3%) will likely recommend testing to patients if applicable. Only a few believe sufficient resources are available to support pharmacogenomics practice (10.5%).

**Table 3:** Pharmacists’ attitude towards pharmacogenomics.

Question (n = 426)	n	(%)
How important do you believe pharmacogenomics is in personalized medicine?		
Slightly important	187	43.9
Moderately important	140	32.9
Extremely important	99	23.2
Do you think that pharmacogenomics should be integrated into the standard pharmacy curriculum?		
Strongly disagree	30	7.0
Disagree	33	7.7
Neutral	178	41.8
Agree	133	31.2
Strongly agree	52	12.2
How confident are you in your ability to apply pharmacogenomics in clinical practice?		
Not confident at all	62	14.6
Slightly confident	250	58.7
Moderately confident	97	22.8
Extremely confident	17	4.0
What are your main concerns regarding the implementation of pharmacogenomics in pharmacy practice? (n = 426) (k = 935)		
Lack of knowledge and training	292	68.5
Cost of genetic testing	260	61.0
Ethical and privacy issues	187	43.9
Integration into existing workflows	66	15.5
Patient acceptance and understanding	130	30.5
How likely are you to recommend pharmacogenomics testing to your patients if it is applicable?		
Very unlikely	18	4.2
Unlikely	45	10.6
Neutral	229	53.8
Likely	90	21.1
Very likely	44	10.3
Do you believe that current pharmacy practice is adequately prepared to incorporate pharmacogenomics?		
No	260	61.0
Yes	166	39.0
To what extent do you agree with the following statements about pharmacogenomics?		
*Pharmacogenomics can improve patient outcomes.*		
Strongly disagree	16	3.8
Disagree	70	16.4
Neutral	82	19.2
Agree	130	30.5
Strongly agree	128	30.0
*Pharmacists should play a key role in pharmacogenomics.*		
Strongly disagree	16	3.8
Disagree	70	16.4
Neutral	117	27.5
Agree	144	33.8
Strongly agree	79	18.5
*Pharmacogenomics testing should be routine in clinical practice.*		
Strongly disagree	47	11.0
Disagree	88	20.7
Neutral	152	35.7
Agree	113	26.5
Strongly agree	26	6.1
*There are sufficient resources available for pharmacogenomics practice.*		
Strongly disagree	123	28.9
Disagree	126	29.6
Neutral	132	31.0
Agree	35	8.2
Strongly agree	10	2.3
*The cost of pharmacogenomics testing is justified by its benefits.*		
Strongly disagree	88	20.7
Disagree	70	16.4
Neutral	201	47.2
Agree	67	15.7
Strongly agree	0	0.0

n = number of participants, k = number of responses in multiple responses questions.

Table 4 reveals that nearly half of the pharmacists (48.4%) have utilized pharmacogenomics information in practice; however, usage is generally infrequent, with most reporting that they rarely incorporate it (31.5%). Key barriers include limited access to genetic testing (74.2%) and lack of reimbursement (64.5%). Additionally, almost half (46.9%) rate their institution as not ready to implement pharmacogenomics routinely. While 40.8% of pharmacists see themselves leading a multidisciplinary team, advising on genetic test interpretation and drug therapy, 34% prefer a supportive role focused on patient information and counseling.

**Table 4:** Pharmacists’ implementation practices towards pharmacogenomics.

Question (n = 426)	n	(%)
Have you ever used pharmacogenomics information in your practice?		
No	220	51.6
Yes	206	48.4
If yes, how frequently do you use pharmacogenomics information in your practice? (n = 206)		
Rarely	134	31.5
Occasionally	36	8.5
Often	36	8.5
What barriers do you face in implementing pharmacogenomics in your practice? (n = 426) (k = 907)		
Lack of access to genetic testing	282	74.2
Insufficient clinical guidelines	185	48.7
Lack of reimbursement for testing	245	64.5
Limited time to stay updated with new information	195	51.3
How would you rate your pharmacy or institution’s readiness to implement pharmacogenomics into routine practice?		
Not ready at all	200	46.9
Slightly ready	179	42.0
Moderately ready	20	4.7
Extremely ready	27	6.3
What role should pharmacists play in the multidisciplinary team regarding pharmacogenomics?		
Leading role in interpreting genetic tests and advising drug therapy	174	40.8
Supporting role in providing information and counseling to patients	145	34.0
Minimal role, limited to providing general advice	94	22.1
No role, other healthcare professionals should handle this	13	3.1

n = number of participants, k = number of responses in multiple responses questions.

### 3.1. Knowledge mean score

Table 5 shows that pharmacists demonstrated overall moderate knowledge in pharmacogenomics, with a mean score of 2.33, corresponding to 46.6% competency. However, significant knowledge gaps remain, particularly in specialized areas such as genetic testing interpretation, ethical considerations, and clinical implications. Key gaps were observed in ethical and legal issues (mean = 1.95), genetic testing and interpretation (mean = 1.99), and clinical implications (mean = 2.06). Pharmacists demonstrated moderate knowledge in pharmacokinetics and pharmacodynamics (mean = 2.80) and received formal education or training in pharmacogenomics (mean = 3.09). Knowledge levels were categorized using modified Bloom’s cutoff criteria, where good knowledge was defined as a score of 75% or higher of the total, moderate knowledge as 50% to 74%, and poor knowledge as 49% or lower. Based on this classification, most pharmacists fell into the moderate or poor knowledge categories, with few achieving a high level of competency in pharmacogenomics.

**Table 5:** Pharmacists’ responses towards pharmacogenomics knowledge.

Statement	Mean	SD	Rank	Level of knowledge
How familiar are you with the term “pharmacogenomics”?	2.47	1.072	3	Inadequate knowledge
Have you received any formal education or training in pharmacogenomics?	3.09	2.000	1	Moderate knowledge
How often do you consult pharmacogenomics resources (e.g., databases, guidelines) in your practice?	2.10	1.228	5	Inadequate knowledge
Have you ever participated in any pharmacogenomics research or projects?	2.09	1.783	6	Inadequate knowledge
Rate your understanding of the following pharmacogenomics concepts:				
*Genetic variations affecting drug response.*	2.42	1.234	4	Inadequate knowledge
*Pharmacokinetics and pharmacodynamics.*	2.80	1.522	2	Moderate knowledge
*Clinical implications of pharmacogenomics.*	2.06	.971	7	Inadequate knowledge
*Genetic testing and interpretation.*	1.99	.935	8	Inadequate knowledge
*Ethical and legal issues in pharmacogenomics.*	1.95	.889	9	Inadequate knowledge
Overall mean score	**2.33**			

### 3.2. Attitude mean score

Table 6 illustrates pharmacists’ positive attitude toward the potential of pharmacogenomics to improve patient outcomes, with a mean score of 3.67. However, attitudes are neutral regarding its inclusion in pharmacy curricula (mean = 3.34) and recommending pharmacogenomics testing (mean = 3.23). Confidence in applying pharmacogenomics (mean = 2.20) and perceptions of adequate resources (mean = 2.26) are low, highlighting key areas needing improvement.

**Table 6:** Pharmacists’ attitude towards pharmacogenomics.

Statement	Mean	SD	Rank	Level of attitude
How important do you believe pharmacogenomics is in personalized medicine?	3.03	1.171	5	Neutral
Do you think that pharmacogenomics should be integrated into the standard pharmacy curriculum?	3.34	1.024	3	Neutral
How confident are you in your ability to apply pharmacogenomics in clinical practice?	2.20	.833	9	Negative attitude
How likely are you to recommend pharmacogenomics testing to your patients if it is applicable?	3.23	.922	4	Neutral
*To what extent do you agree with the following statements about pharmacogenomics?*				
*Pharmacogenomics can improve patient outcomes.*	3.67	1.175	1	Positive attitude
*Pharmacists should play a key role in pharmacogenomics.*	3.47	1.085	2	Neutral
*Pharmacogenomics testing should be routine in clinical practice.*	2.96	1.076	6	Neutral
*There are sufficient resources available for pharmacogenomics practice.*	2.26	1.037	8	Negative attitude
*The cost of pharmacogenomics testing is justified by its benefits.*	2.58	.987	7	Neutral
Overall mean score	**2.97**			

### 3.3. Practice mean score

Table 7 reveals that pharmacists report a good level of practice regarding their role in a multidisciplinary team for pharmacogenomics (mean = 2.38). However, the frequency of pharmacogenomics information (mean = 1.35) and institutional readiness for routine implementation (mean = 1.17) reflect poor practices.

**Table 7:** Pharmacists’ practice towards pharmacogenomics.

Statement	Mean	SD	Rank	Level of practice
Have you ever used pharmacogenomics information in your practice?	1.97	1.001	2	Moderate practice
If yes, how frequently do you use pharmacogenomics information in your practice?	1.35	.478	3	Bad practice
How would you rate your pharmacy or institution’s readiness to implement pharmacogenomics into routine practice?	1.17	.521	4	Bad practice
What role should pharmacists play in the multidisciplinary team regarding pharmacogenomics?	2.38	.545	1	Good practice
Overall mean score	**1.73**			

### 3.4. Associations between socio-demographic factors and pharmacists’ levels of knowledge, attitude, and practice towards pharmacogenomics

Table 8 shows that age, years of experience, and type of pharmacy practice are significantly associated with knowledge, attitude, and practice levels in Pharmacogenomics. Gender is significantly associated with knowledge and attitude but not with practice. Education level is significantly associated with knowledge and practice but not with attitude. These results highlight key socio-demographic factors influencing pharmacists’ engagement with Pharmacogenomics.

**Table 8:** Socio-demographics and pharmacogenomics: knowledge, attitude, and practice associations.

Demographics	Association with Knowledge Level	*P*-value (Knowledge)	Association with Attitude Level	*P*-value (Attitude)	Association with Practice Level	*P*-value (Practice)
Age	Significant	.001*	Significant	<.001*	Significant	.004*
Gender	Significant	.047*	Significant	.018*	Not Significant	.108
Education Level	Significant	.002*	Not Significant	.086	Significant	<.001*
Years of Experience	Significant	<.001*	Significant	.003*	Significant	<.001*
Type of Pharmacy Practice	Significant	<.001*	Significant	<.001*	Significant	<.001*

* indicates that the value is significant because it is under 0.05.

## 4. Discussion

### 4.1. Main findings

This study comprehensively assesses pharmacists’ KAP regarding pharmacogenomics in Saudi Arabia, highlighting significant preparedness gaps and suggesting areas for improvement. Most pharmacists recognize the relevance of pharmacogenomics in advancing personalized medicine, but they demonstrate inadequate knowledge levels, particularly in specialized areas such as the interpretation of genetic testing, clinical implications, and ethical considerations. Although 52.3% reported some pharmacogenomics education, most indicated basic familiarity with key concepts. Furthermore, most pharmacists were unsure of how to apply pharmacogenomics in practice, with low confidence in their abilities to implement it effectively in patient care. Socio-demographic variables, such as age, years of experience, and pharmacy practice setting, were significantly associated with pharmacists’ KAP, suggesting that professional exposure influences pharmacogenomics engagement. These findings indicate the need for an integrated approach to strengthen pharmacists’ competency in this emerging field.

### 4.2. Main discussion

The study findings reveal a significant gap between pharmacists’ perceived importance of pharmacogenomics and their current readiness to apply it in practice, acknowledging that pharmacogenomics remains an emerging field with limited routine clinical applications at present. This highlights the need for gradual, structured integration efforts, and realistic expectations regarding its implementation. While most participants acknowledged the relevance of pharmacogenomics in personalized medicine, their overall knowledge levels were inadequate, particularly in areas such as genetic test interpretation and clinical application. This aligns with previous studies conducted in North America, Europe, and the Middle East, where pharmacists expressed support for pharmacogenomics but reported limited training and low confidence in its practical application.^[24]^ For instance, a study in Saudi Arabia found that only 29.8% of pharmacists possessed good knowledge of pharmacogenomics, with significant knowledge gaps among those lacking formal training.^[25]^ Similarly, research in the United Arab Emirates has shown that community pharmacists exhibit limited knowledge about pharmacogenomic testing, with workload factors, such as the number of patients and prescriptions they handle daily, influencing their knowledge and attitudes.^[12]^ These findings underscore the need for enhanced educational initiatives to equip pharmacists with the necessary competencies to integrate pharmacogenomics into patient care effectively.

A key challenge identified in this study was the limited exposure to pharmacogenomics education, with only 52.3% of pharmacists receiving formal training, primarily during their university coursework. This is consistent with findings from Canada and UK, where pharmacy curricula have been criticized for insufficient pharmacogenomics content, resulting in knowledge gaps that persist into professional practice.^[26]^ A lack of structured postgraduate education and continuing professional development (CPD) programs in pharmacogenomics further exacerbates this issue. Pharmacists in many countries, including South Korea and Japan, have difficulty accessing updated pharmacogenomics guidelines and resources, highlighting the urgent need for integrated pharmacogenomics training at both undergraduate and postgraduate levels.^[27]^

Beyond education, another significant barrier was the limited access to genetic testing services reported by 74.2% of participants. This challenge has been widely documented in international research, with studies in the United States and Australia indicating that the availability and affordability of pharmacogenomic tests are critical determinants of clinical uptake.^[28]^ Pharmacists often lack the institutional support or authority to request genetic tests, which limits their ability to apply pharmacogenomics in practice. Additionally, 64.5% of pharmacists identified the lack of reimbursement models as a significant obstacle, mirroring findings from research in Europe and Asia, where the absence of structured reimbursement systems has hindered pharmacogenomics adoption.^[29]^ Without financial incentives or insurance coverage for genetic testing, healthcare providers and patients remain hesitant to utilize pharmacogenomic services, further slowing their integration into clinical practice.

This study also found that socio-demographic factors such as age, years of experience, and practice setting significantly influenced pharmacists’ knowledge and attitudes toward pharmacogenomics. Similar associations have been reported in studies from Malaysia and Egypt, where pharmacists working in hospital and clinical settings demonstrated higher pharmacogenomics competency and a greater willingness to integrate it into practice than those in community pharmacy settings.^[28,30]^ This suggests that exposure to a multidisciplinary healthcare environment, where pharmacists frequently collaborate with physicians, geneticists, and other specialists, enhances their familiarity with and confidence in pharmacogenomics. In contrast, pharmacists working in community settings, where pharmacogenomic testing and clinical decision-making are less commonly integrated, may have fewer opportunities to engage in this field. These findings emphasize the need for targeted educational interventions tailored to different pharmacy practice settings, ensuring that pharmacists in all roles receive adequate training in pharmacogenomics.

Given these barriers, it is crucial to implement comprehensive educational and institutional strategies to support the integration of pharmacogenomics into pharmacy practice. Pharmacy education curricula should be revised to include more extensive training in pharmacogenomics, incorporating practical case studies, exercises in genetic test interpretation, and exposure to real-world applications. Additionally, structured CPD programs should be developed to provide pharmacists with ongoing opportunities to enhance their pharmacogenomics competency. International models, such as those in the Netherlands and the United States, have shown that embedding pharmacogenomics into CPD frameworks significantly improves pharmacists’ confidence and engagement in this area.^[31]^

Furthermore, institutional and policy-level changes are necessary to facilitate pharmacists’ roles in pharmacogenomics. As the field evolves, expanding access to genetic testing, integrating pharmacogenomic decision-support tools into electronic prescribing systems, and establishing reimbursement models may support its gradual adoption. Collaborative efforts among regulatory authorities, healthcare institutions, and pharmacy organizations will be necessary to define pharmacists’ responsibilities in pharmacogenomics and support their involvement in patient-centered genetic medicine.

### 4.3. Strengths and limitations of the study

The study’s cross-sectional design and diverse sample from multiple practice settings enhance its generalizability, offering valuable insights into pharmacists’ perspectives across Saudi Arabia. The use of a validated survey instrument, refined through expert consultation and pilot testing, bolsters the reliability of the data collected. However, the snowball sampling method, while helpful in reaching a wide range of participants, may introduce selection bias, as those already interested in pharmacogenomics may have been more likely to participate. The reliance on self-reported data poses a risk of response bias, where participants may overestimate or underestimate their knowledge and attitudes. The findings also do not account for potential regional differences within Saudi Arabia, which could influence pharmacists’ exposure to pharmacogenomics resources and training opportunities. Additionally, the interpretation of gender-based differences in knowledge and attitudes should be approached cautiously, given the unequal representation of males and females in our sample (72.5% male). Future research with a more balanced gender representation may be needed to explore gender-specific associations with pharmacogenomics knowledge and attitudes accurately.

## 5. Conclusion

This study highlights significant gaps in pharmacists’ knowledge and readiness for pharmacogenomics in Saudi Arabia. While most pharmacists recognized its importance, their knowledge was inadequate, particularly in the interpretation of genetic tests and their clinical applications. Only 52.3% had formal training, and 40.6% had never consulted pharmacogenomics resources. Confidence in applying pharmacogenomics was low, with only 4% feeling highly confident. Key barriers included limited access to genetic testing (74.2%), lack of reimbursement (64.5%), and insufficient institutional support. Socio-demographic factors such as age, experience, and practice setting significantly influenced pharmacists’ engagement with pharmacogenomics. Targeted education, clinical guidelines, and policy reforms are needed to enhance pharmacists’ competency and support the gradual and evidence-based integration of pharmacogenomics into pharmacy practice as the field evolves.

## Acknowledgments

The author would like to acknowledge Deanship of Graduate Studies and Scientific Research, Taif University for funding this work.

## Author contributions

**Conceptualization:** Mohammed S. Alharthi.

**Data curation:** Mohammed S. Alharthi.

**Formal analysis:** Mohammed S. Alharthi.

**Funding acquisition:** Mohammed S. Alharthi.

**Investigation:** Mohammed S. Alharthi.

**Methodology:** Mohammed S. Alharthi.

**Software:** Mohammed S. Alharthi.

**Writing – original draft:** Mohammed S. Alharthi.

**Writing – review & editing:** Mohammed S. Alharthi.
